# Atmospheric Pressure Plates

**Published:** 1852-01

**Authors:** J. P. Ullrey


### ATMOSPHERIC PRESSURE PLATES.

*Address of Dr. J. P. Ullrey, read before the Mississippi Valley Association of Dental Surgeons, held in Louisville September* 11*th*, 12*th*, *and* 13*th*, 1851.

Mr. President, and Gentlemen of the Mississippi Valley Association of Dentists—At our last annual meeting, held in Cincinnati, I was appointed to prepare and read an article before you, on the subject of Atmospheric Pressure, as applied to, and retaining in the mouth, artificial teeth. I feel that this duty should have fallen on some member of the profession who was better qualified, by a more extensive practice than I have enjoyed; for it is a subject which needs more light shed upon it than has, as yet, come to the knowledge of the profession generally, and especially to your speaker.

True, this is no new subject to the profession, yet there are but few in practice that ever attempt to put up a set of teeth on the atmospheric principle, and the few that make the effort frequently find that the work does not hold as well as they would wish; at least, such is my experience.

It would be wasting your time for me to trace out all who have laid claim to the invention of suction, or atmospheric pressure, as applied to the holding and keeping in place a set of upper teeth, so that the patient can masticate food with them; and it is useless for me to try and seek out the first inventor really, for it would be of little service now to the profession.

The great question now to decide is, can atmospheric pressure be applied to the retention of artificial teeth in the mouth. This point I deem entirely settled. It can be done, and time, the greatest test of all human inventions, has given testimony in behalf of the assertion I have made, that "it can be done."

And I need not tell these intelligent gentlemen before me, that when it is done well, that our patients are not only surprised but well satisfied. But the most important consideration is, what is the best manner of making use of the atmospheric pressure in retaining whole or parts of sets of teeth in the mouth. This part of my subject, (and, in truth, the only part which needs any consideration or explanation,) has given me some trouble, and I really hesitate to give my experience on this subject to my brother dentists, for fear of wasting their time, which might be more profitably spent. However, with the indulgence of the brethren, I will add some of my experience to that which they are already familiar with. In making atmospheric plates, I have tried nearly all the different kinds and shapes of air chamber that I have heard of, or could think of myself; and first, I will mention the air chambers made by raising small elevated points on the plaster cast, one on each side, just inside of the straps, and situated near the points occupied by the first large molar. This plan, at first thought, seemed to be the thing I wanted, but I was not so well satisfied after my patient made a trial of the work. Secondly, I tried a large chamber, a half circle in the middle of the plate, about half an inch from the center of the alveolar ridge, the chamber being one line deep, square edges, length near one inch. Thirdly, tried two plates by raising the alveolar ridge about one line in the center, tapering down to nothing on each side, under the second bicuspids; width one-fourth of an inch. These modes, with variations, such as giving more or less length, and depth, or width, to my air chambers, I have tried to my satisfaction, and have finally abandoned them and fallen back upon the plain, narrow, close fitting plates, believing that when a plate is well fitted, that it answers a better purpose than the cavity plate. So they have proven in my practice, so far as full upper sets of teeth are concerned. I have also experimented with small sets, or rather parts of sets of teeth, with the hope of being able to dispense with clasps or bands, and thereby save many good teeth for my patients; and I have the satisfaction of being able to report success with small pieces of work, such as setting one and two teeth on plates held in their place by atmospheric pressure, and retained so firm that my patients could masticate with them without inconvenience. I will here remark, that in my small plates I have made an air chamber, some by elevating my plate a little in the center, others by making a close fit and then perforating it with some four or five small holes, and soldering a thin piece of plate slightly elevated over that part. In small pieces, as well as full sets of teeth, I always extend my plate as far over the outside of the gum as possible. In preparing for an atmospheric plate I make a practice of taking a copy of the mouth with plaster, in all cases where it is practicable, in order that I may secure a perfect fit. With parts of sets I am compelled to use wax for the want of a better substance. The most important feature is, to get a perfect copy of the mouth, and keep it up throughout all your manipulations. My mode of getting up sets is, first, after oiling the plaster or wax moulds, I fill it with plaster mixed to the consistence of thick cream, shaking the mould moderately that it may fill perfectly in every part, then let it get perfectly dry before separating. The next step is, to get up the metalic casts. In this, perhaps, I differ some from the general practice.

I find the harder my metal is the more perfect I can strike up the plate. For my female casts I use type metal or lead mixed with block tin, after melting and cleansing the metal from dross, pour it into a sheet-iron cup, and when the edges begin to cool, dip the plaster model into it and hold it firm and steady until it becomes hard. After carefully breaking out the plaster, (to give draft and shape to the male casts,) I fill round with clay or moulding sand, then with a camel's hair brush put a light coat of prepared chalk mixed with water on the female cast to prevent the male cast from sticking. When perfectly dry, melt the zinc for the male cast, and pour into the mould thus prepared. After much experience, I have adopted this as the most perfect method of obtaining a fac simile of the mouth. This is my personal experience, and hoping that there will be a full expression of the opinions of all present, I submit these remarks, with my thanks to my brother dentists for their courtesy and attention.

